# Editorial: Bacterial surface polymers

**DOI:** 10.3389/fcimb.2024.1415799

**Published:** 2024-04-25

**Authors:** Tarcisio Brignoli, Marco Spinsanti, Maisem Laabei, Seána Duggan

**Affiliations:** ^1^ Department of Biosciences, Università degli Studi di Milano, Milan, Italy; ^2^ GSK, Siena, Italy; ^3^ School of Cellular and Molecular Medicine, University of Bristol, Bristol, United Kingdom; ^4^ The Medical Research Council Centre for Medical Mycology at the University of Exeter, Exeter, United Kingdom

**Keywords:** bacterial surface polymers, capsule, lipopolysaccharide, O-antigen, biofilm

The bacterial surface is an essential structure that mediates all interactions between a bacterial cell and its immediate extracellular environment. Bacterial polymers are integral components of this surface architecture, which function in a multitude of cellular processes including cell division and spatial organization, molecular trafficking, protection from antimicrobial agents, and interaction with and evasion of the host (Gao et al., 2024; Brown et al., 2013; Sabnis et al., 2018; Brignoli et al., 2022). Their importance is highlighted by the stringent regulation of their synthesis, complex molecular machinery driving their biosynthesis, and the metabolic costs invested in their production (Whitfield et al., 2020; Galinier et al., 2023). Given the importance of surface polymers for bacterial physiology, it is not surprising that many prophylactic or therapeutic strategies, such as vaccines or antibiotics, target surface polymers (Zahlanie et al., 2014; Tennant et al., 2016; Zhou et al., 2022). With this Research Topic we collated research and review articles on bacterial surface polymers, and this article reports the main findings and perspectives of each accepted manuscript.

The outermost bacterial surface polymer is the capsule, which is typically made of polysaccharide chains covering the outer layer of the cell. This structure is not essential or expressed across all strains, however when present it is often part of the various immune evasion systems employed by bacterial pathogens, that can limit complement and antibody deposition and impair phagocytosis (Nanra et al., 2013; Brignoli et al., 2019; Kamuyu et al., 2022). Variation in the production of capsule can result in different virulence capacities even within clinical isolates of the same pathogen, exemplified by hypervirulent *Klebsiella pneumoniae* which displays increased capsule formation (Russo and Marr, 2019; Choby et al., 2020). The work of Wang et al., investigates the molecular mechanisms leading to higher capsule production in hypervirulent *K. pneumoniae*. They show how three main serotypes of hypervirulent *K. pneumoniae* (K1, K2, and K64) are characterized by different alleles of the *wcaJ* gene (*K1wcaJ*, *K2wcaJ*, and *K64wcaJ*) which encodes the glycosyltransferase initiating capsule synthesis. The authors demonstrate that the three alleles of *wcaJ* are not interchangeable: complementation of a K1 Δ*wcaJ* strain was demonstrated only with the *K1wcaJ* and *K2wcaJ* alleles, but not with *K64wcaJ*. In fact, these mutants produced different amounts of capsule, with effects on biofilm production, phagocytosis by macrophages, serum resistance and infection severity in a *Galleria mellonella* infection model. Moreover, the impact of the transcriptional regulator RmpA on hypervirulent *K. pneumoniae* capsule production was evaluated. Here, the *rmpA* sequence was conserved among the different serotypes, and deletion of *rmpA* results in lower capsule production.

In Gram-negative bacteria, lipopolysaccharide (LPS) sits beneath the capsule and forms the main component of the extracellular leaflet in the outer membrane (Bertani and Ruiz, 2018). LPS molecules are glycolipids, composed of three structural domains: lipid A, the core oligosaccharide, and the O-antigen. Lipid A and the core oligosaccharide are usually conserved within bacterial species, while the distal extended polysaccharide that compose the O-antigen is highly variable. LPS can induce a strong inflammatory response in the host, which can result in toxic shock syndrome. It is essential that pharmaceutical products are LPS-free, but the gold standard for LPS detection (LAL-assay) still relies on the blood of the horseshoe crab, an endangered species (Schneier et al., 2020). Hatlem et al. address this issue by evaluating potential LPS-binding proteins. Surprisingly, they discovered that a modified coiled-coil sequence derived from the yeast transcription factor GCN4 can bind to LPS with high affinity. Further experiments showed that the trimeric variant of this protein, GCN4-pII, also binds to the lipid A of a broad range of Gram-negative species. Detection of LPS using the GCN4-pII showed similar, if not increased, sensitivity compared to the LAL-assay. Moreover, GCN4-pII not only binds to LPS but also dissolves LPS micelles in solution, providing proof-of-concept for future potential applications.

Another biotechnological application described in this Research Topic is the use of O-antigens as vaccine components. The O-antigen is the most variable component of LPS, and it is composed of a variable number of repeating oligosaccharide units. The O-antigen structure varies by composition of the sugars in the backbone, as well as further modifications such as O-acetylation and glucosylation (Micoli et al., 2014; Ravenscroft et al., 2015). These differences can have an impact on the immunogenicity and the cross-reactivity of the antigen. The work of Gasperini et al. investigated these aspects of the O-antigens produced by different *Salmonella enterica* serovars. The immunogenicity of different O-antigen structures was evaluated using GMMA, outer membrane vesicles released from genetically engineered Gram-negative bacteria, which display the O-antigen in its natural outer membrane context. Bactericidal activity of sera raised against different serovars showed a certain degree of heterologous killing for all O-antigens. The *S. Paratyphi A* GMMA were selected for eliciting the highest heterologous bactericidal activity, and further analysis demonstrated that the O-antigen glucosylation and O-acetylation were the major determinants of cross-reactivity. This work presents useful findings to estimate the coverage of O-antigen based *Salmonella* vaccines.

Finally, the review of Perry and Tan describes the bacterial biofilm in the context of the human host. This detailed review illustrates where biofilms are found in the human body, in the context of both health and disease environments, as well as the causative species or taxa. The authors report the factors that modulate tissue-associated biofilm development, including adhesion factors, composition of the extracellular matrixes, and environmental conditions. In addition, the impact that tissue-associated biofilms have on human health is also addressed. Despite the breadth of information covered, the field still has many knowledge gaps such as the prevalence of biofilms in commensal states and the impacts of interspecies interactions and polymicrobial biofilms. Identifying these issues is the first step to addressing them, but progress will only follow the development of relevant experimental models.

In conclusion, the articles and review collected in this Research Topic highlight the importance of deepening our knowledge of the surface polymers produced by bacteria to better understand their regulation and their impact on health and disease. This will in turn provide new information for the development of novel antimicrobial strategies.

## Author contributions

TB: Writing – original draft, Writing – review & editing. MS: Writing – review & editing. ML: Writing – review & editing. SD: Writing – review & editing.
